# Mapping brain-wide activity networks: brainways as a tool for neurobiological discovery

**DOI:** 10.1038/s41386-025-02105-3

**Published:** 2025-04-22

**Authors:** Ben Kantor, Keren Ruzal, Inbal Ben-Ami Bartal

**Affiliations:** 1 School of Psychological Sciences, Tel-Aviv University, Tel Aviv, Israel; 2 Sagol School of Neuroscience, Tel-Aviv University, Tel Aviv, Israel

**Keywords:** Microscopy, Empathy

## Abstract

Identifying brain-wide neural circuits and targeting these areas for neuropharmacological interventions are significant challenges in contemporary neuroscience. Traditional methods for registering and quantifying fluorescence in brain slices are labor-intensive and struggle to extract functional insights from complex datasets. To address these challenges, we introduce Brainways—an AI-based, open-source software that streamlines neural network identification from digital imaging to network analysis. Brainways facilitates neurobiological research by enabling automatic registration of coronal brain slices to any 3D brain atlas, along with precise quantification of fluorescent markers, such as activity markers and tracers, across brain regions. Brainways incorporates advanced statistical tools to identify neural patterns and functional networks associated with specific experimental contrasts. Trained on rat and mouse brain atlases, Brainways achieves over 93% atlas registration accuracy. The software also allows users to easily adjust the automatic registration through a user-friendly interface for enhanced accuracy. We present two experiment analyses demonstrating Brainways’ capabilities. The first replicates and extends findings from a prior experiment on pro-social behavior in rats, wherein rats learned to free a trapped cagemate from a restrainer under ingroup and outgroup social conditions. Using Brainways, we analyzed approximately 300 times more tissue area than in our previous manual approach. The second experiment utilizes Multiplex RNAscope imaging for whole-brain registration, enabling combined quantification of cell type expression and activity markers. These analyses highlight Brainways’ ability to link specific cell types and their activity to task conditions, providing detailed neural insights. Brainways offers a rapid and accurate solution for large-scale neurobiological projects, creating new opportunities to understand neural networks underlying complex behaviors.

## Introduction

Fluorescent tagging for immediate early genes (IEG) has been increasingly used to identify functional networks that participate in complex behaviors [1–4]. While this strategy is not without caveats, such as low temporal resolution and the use of an indirect index for activity, it has led to important insights and is being globally adopted. The advantage of this approach is that it provides unbiased data-driven hypotheses for follow-up manipulations on specific neural projections and subpopulations. Furthermore, activity tagging can be combined with other markers of cell identity, such as cell type, receptors, or a tracer, to provide enhanced activity-identity mapping of subpopulations of interest.

While this approach shows promise, several obstacles have impeded progress. First, quantification and analysis are difficult, especially for high-resolution datasets. Analyzing fluorescence in histological images requires registration to a common reference brain atlas, followed by quantification of fluorescence levels or number of marker-positive cells in each brain region of interest (ROI). Due to a lack of available user-friendly tools to perform registration and quantification in a fast, streamlined, and accurate manner, this process is often performed manually, limiting the scope of the investigation to specific ROIs or a low sampling rate in order to reduce the quantification timeline. Unlike manual analysis, which requires expertise, is labor-intensive and prone to inter-rater variability, automatic registration and quantification provides efficient and reliable analysis of entire digital sections. Another substantial challenge lies in analysis and interpretation of brain-wide quantification, especially as the bar continues to rise in terms of resolution and cell specificity. Tools that use brain-wide activity mapping data to generate neural network “connectomes” are needed to bridge this gap.

To this end, we developed Brainways, an open source, rapid, and user-friendly AI-based software that provides an automated pipeline for the analysis of fluorescence tagging on coronal sections. Brainways aims to address these challenges and provide a platform for collaboration between labs using different model organisms for studying the brain. Brainways provides an easy way to perform automated registration, quantification, and statistical analysis of histological brain slices from a whole experiment. Brainways provides an easy-to-use graphical user interface (GUI), performs cell detection and quantification, maps detected cells to registered ROIs, and performs statistical comparisons between subjects in different experimental conditions, providing a short turn-around from slide scanning to brain-wide quantification and analysis.

The Brainways GUI offers an interface for visualizing and refining automatic registrations, reviewing cell detection outcomes, and adjusting detection parameters. Additionally, it provides graphical visualizations of neural patterns associated with contrasts between experimental conditions. Automatic registration utilizes an innovative deep learning algorithm, trained jointly on both mouse and rat brain slices, which enhances the accuracy of each modality through cross-species learning. For further details on the implementation of Brainways’ functionalities, refer to Supplementary Note 1.

Brainways presents enhanced functionality compared to existing software solutions. Several algorithms exist for histological slice registration [5–7], which are mostly suitable for mouse brains. Additionally, all of these algorithms require integration with other software to complete the analysis, resulting in a cumbersome workflow and often requires programming experience. Brainways offers functional insights on the brain-wide activity of a wide range of behaviors of interest across species, accessible to users at any level working with coronal slices, which are used by many labs for their high accessibility and cost-effectiveness.

Lightsheet microscopy, often combined with tissue-clearing methods such as iDISCO [8], is emerging as a powerful approach for capturing volumetric images of whole brains. Several automatic registration algorithms have been developed specifically for this modality [3, 9–11], offering valuable whole-brain imaging capabilities. However, these pipelines are not well-suited for the many neuroscience applications that rely on coronal brain slices, which remain widely used due to their cost-effectiveness, accessibility, and compatibility with established staining and molecular labeling techniques. Yet, physical sectioning can introduce tearing, warping, and significant elastic deformations—challenges largely absent in intact cleared tissue—and tissue processing workflows often disrupt the sequential order of slices during staining (i.e. free-floating methods), making manual reordering time-consuming and requiring anatomical expertise.

Brainways addresses these challenges by providing a dedicated solution for coronal slice registration that is robust to non-rigid deformations and missing sequential context. In addition to its advanced AI-based automatic registration, Brainways offers an integrated GUI for intuitive visual inspection, fine-tuning, and manual correction of registrations. This functionality is critical for accurate alignment of histological datasets, where automated algorithms alone may struggle with the distortions introduced by tissue processing. Furthermore, Brainways enables analyses such as cell detection and statistical evaluations that are not readily supported by lightsheet pipelines. By filling this methodological gap, Brainways serves as a useful and accessible tool for neuroscientists who rely on the versatility and familiarity of section-based techniques, reinforcing the ongoing importance of histological approaches in neuropharmacological studies.

To assess the validity and reliability of Brainways’ quantification across different scenarios, we present analyses conducted using Brainways on data from two experiments. The first is a re-analysis of a manually quantified dataset previously published by our group [12]. This dataset consists of rat brain slices stained with the immediate early gene marker c-Fos to measure neural activity, with the study aiming to map neural activity related to prosocial motivation. In this re-analysis, the main findings from the original publication were reliably reproduced, despite differences in quantification methods and the broader coverage of neural tissue compared to the original quantification. With Brainways, re-analysis of the dataset required approximately two weeks, compared to the multi-month manual effort of the original study. This reduction in processing time owes largely to Brainways’ automated slice registration, integrated automatic cell detection and statistical analyses, and user-friendly interface, which significantly decrease the need for labor-intensive manual segmentation and repeated complex data handling.

The second analysis examines an experiment using Multiplex RNAscope imaging, showcasing Brainways’ ability to perform whole-brain registration and integrated quantification of cell type expression alongside activity markers. This approach enabled us to link specific cell types and their activity to task conditions, offering detailed neurobiological insights. The use of Brainways in this context underscores its potential to advance neuropharmacological research and facilitate the exploration of novel neurobiological mechanisms underlying complex behaviors.

In summary, Brainways is a powerful tool that facilitates the discovery of novel neurobiological mechanisms underlying complex behaviors. The software is freely available on GitHub (https://github.com/bkntr/brainways), accompanied by comprehensive documentation and instructional video demonstrations.

## Materials and methods

### Brainways

**Fig. 1:**
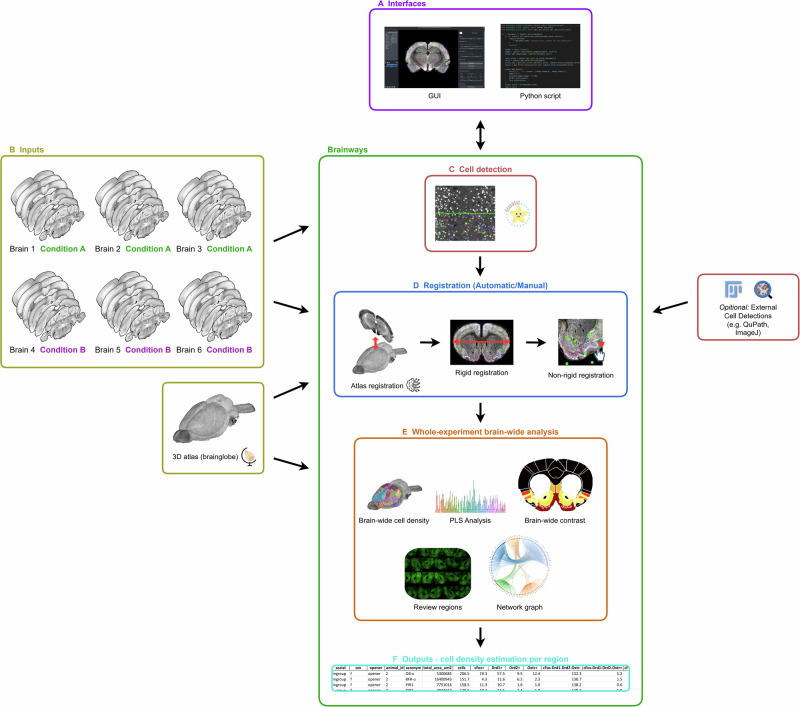
Overview of the brainways workflow. **A** Brainways provides a user-friendly graphical user interface (GUI) for non-programmers and a Python API for advanced users to customize and create complex applications. **B** Inputs to Brainways include coronal slices from multiple subjects, their respective experimental conditions, and a 3D brain atlas via the Brainglobe API. **C** Brainways identifies cells in the registered brain slices using the StarDist algorithm, with the option to incorporate external cell detections from software such as QuPath or ImageJ for those with existing detection pipelines. **D** Brain slices are registered to the atlas both automatically and manually. Initially, images are aligned to the Anterior-Posterior axis of the 3D atlas using a dedicated AI algorithm. The slices are then roughly matched to the atlas through translation, scaling, and rotation, followed by elastic deformation to correct non-rigid deformations. **E** Brainways performs statistical analyses, including brain-wide cell density calculations per region, partial least squares (PLS) analysis, network graph construction, contrast analysis between experimental conditions, and visual review of registered brain regions. Apart from the network graph, all figures presented here were generated directly in Brainways. The visual rendering of the network graph was performed using the graph-tool library (Peixoto, 2017) based on a network graph file produced in Brainways. **F** The output from Brainways includes cell density per region, presented in a detailed and comprehensive tabulated format.

Brainways comprises multiple modules that enable a seamless progression from digital images to brain-wide statistical analysis and positive cell counts across an entire cohort of subjects (Fig. 1). Users can access these modules via either the Python API or a user-friendly GUI, accommodating both advanced users and those with limited coding experience. Each module contributing to the comprehensive functionality of Brainways is detailed in Fig. S1 and Supplementary Note 1.

### Experimental design

The methods used to obtain the data used in the experiments have been described in detail in [12]. Thus, they are briefly described below.

#### Animals

Rat studies described in the first experiment were performed according to protocols approved by the Institutional Animal Care and Use Committee of the University of California, Berkeley. Rats were kept on a 12-h light-dark cycle and received food and water ad libitum. In the first experiment, a total of 83 rats were tested across all experiments. Adult pair-housed male Sprague Dawley (SD) rats (age p60–p90 days) were used as free and trapped ingroup rats (Charles River, Portage, MI). Adult male Long–Evans rats were used as trapped outgroup rats (LE, Envigo, CA). The second rat experiment was conducted in Tel-Aviv, and performed in accordance with protocols approved by the institutional animal ethics committee at Tel-Aviv University, Israel. A total of 30 rats were tested across experiments. Adult male and female LE rats were used as free and trapped ingroup rats. Adult male SD rats were used as trapped outgroup rats. All rats ordered were allowed a minimum of 5 days to acclimate to the facility prior to beginning testing.

#### Helping Behavior Test

The HBT was performed as previously described [13]. During the HBT, a free rat was placed in an open arena containing a rat trapped inside a restrainer. The free rat could help the trapped rat by opening the restrainer door with its snout, thereby releasing the trapped rat. One-hour sessions were repeated daily over a 2-week period. At 40 min, the door was opened half-way by the experimenter; this was typically followed by the exit of the trapped rat and was aimed at preventing learned helplessness. Door opening was counted as such when performed by the free rat before the half-way opening point. Rats that learned to open the restrainer and consistently opened it on the last three days of testing were labeled ’openers’. In the “separated” HBT, a plexiglass wall divided the arena into two compartments, thus preventing social contact following the release of the trapped rat. Two social conditions were tested: ingroup and outgroup. In the ingroup condition, the two rats (free and trapped) are from the same strain and have lived together in the same home cage for at least two weeks before the experiment. In the outgroup condition, the two rats are from different strains and were introduced to each other for the first time in the first session of the HBT. The free rat was introduced to the same stranger rat in the restrainer throughout the experiment. On the last day of testing, the restrainer was latched shut throughout the 60-minute session, and rats were perfused immediately following behavioral testing.

#### Immunohistochemistry

On the last day of testing, the animals were sacrificed within 90 min from the beginning of the session, at the peak expression of the early immediate gene product c-Fos. Brains were extracted following perfusion, frozen, sliced at 40 µm, and stained for c-Fos. The sections were stained for c-Fos using rabbit anti-c-Fos antiserum (ABE457; Millipore, 1:1000; 1% NDS; 0.3% TxTBS) with Alexa Fluor 488 conjugated donkey anti-rabbit antiserum (AF488; Jackson, 1:500; 1% NDS; 0.3% TxTBS). The sections were further stained in DAPI (1:40,000). Details of the imaging and manual acquisition methods can be found in the original publications [12]. Immunostained tissue was imaged at 10× using a wide-field fluorescence microscope (Zeiss AxioScan) and processed in Zen software.

#### Multiplex RNAscope

Brains from LE rats tested in the “separated” HBT in each social condition described above were used for fluorescent in situ hybridization (ISH) essays (ingroup, outgroup, and naïve baseline, *n* = 6 each, half males and half females in each group). In order to reduce variability associated with door-opening in this relatively small sample, “openers” were selected for the ingroup and “non-openers” for the outgroup. As above, in the final session, restrainers were latched so that neural activity reflects an hour in the presence of a trapped rat. Rats were transcardially perfused with 1XPBS after the last HBT session. The brains were then snap-frozen and stored at −80 °C until cryosectioning at 18 µm. Coronal slices were collected on Superfrost Plus slides (Thermo Scientific) from each brain (4.20 mm, 1.56 mm, 0.00 mm, −2.76 mm, −3.36 mm, −4.44 mm, and −5.64 mm from bregma) and underwent processing for single-molecule fluorescent in situ hybridization (smFISH) assay. Slides were pre-treated with RNAscope Protease III reagent. smFISH was performed on slides using the RNAscope LS Multiplex Reagent Kit (Advanced Cell Diagnostics) and LS 4-Plex Ancillary Kit and Multiplex Reagent Kit on a robotic staining system (Leica BOND-III). RNAscope probes were Fos (ACD, 403598), *DrD2* (ACD, 315648), *DrD1*a (ACD, 317038). Images were acquired on a Vectra Polaris Automated Quantitative Pathology Imaging System (Akoya Biosciences) at 20× magnification. Cellular and subcellular detection was conducted using QuPath software and implemented into Brainways for image registration and brain-wide quantification.

### Dataset curation

To perform the registrations for the following analyses, four annotators blind to task condition each received a portion of the original dataset. Annotators used Brainways GUI to perform automatic registrations for each slice, followed by manual fit adjustments when they perceived a mismatch between the atlas and the digital scan. The complete dataset was then reviewed by a fifth annotator to create the final registrations. For the first experiment, all usable coronal slices (i.e., those without significant tissue tearing and in focus) were included, resulting in an average of approximately 19 slices per brain (mean: 18.71, SD: 6.72). In the RNAScope experiment, approximately 6 slices per brain were processed (mean: 5.80, SD: 1.08).

## Results

To demonstrate Brainways’ practical capabilities, we present two experiment analyses that illustrate its effectiveness across diverse experimental contexts.

### Comparing brainways-assisted quantification to manual quantification

**Fig. 2:**
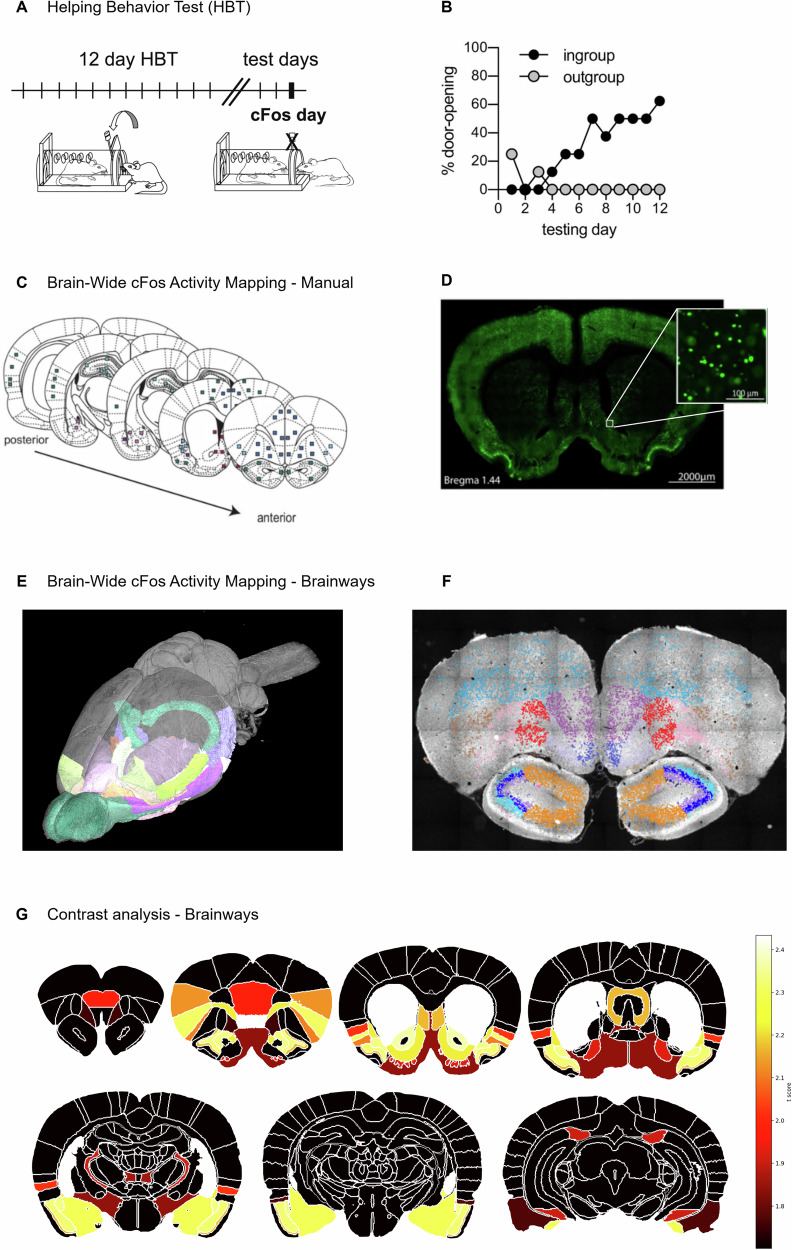
The Brainways pipeline provides a contrast analysis between task conditions. **A** Diagram of the Helping Behavior Test. **B** Rats released ingroup members but not outgroup members, measured by door-opening over the test days. **C**, **D** Diagram of manual acquisition of c-Fos activity mapping, as was performed in Ben-Ami Bartal et al. (2021). The slices were manually matched to the Waxholm Space Rat Atlas (Papp et al., 2014). From these slices, 84 regions of interest were manually extracted followed by counting c-Fos activated cells in each region. **E**, **F** Brain-wide c-Fos activity mapping as automatically acquired in the Brainways pipeline. All slices were automatically registered to a 3D version of the Waxholm Space Rat Atlas (Osen et al., 2019) to obtain an activity mapping with much higher spatial resolution compared to the previous method. **G** ANOVA, post hoc contrast analysis between experimental conditions as visualized using Brainways. (Figures **E**–**G** were generated in Brainways).

To investigate the validity of Brainways and demonstrate its advantages over manual quantification methods, we present a Brainways-assisted quantification of a dataset previously published by our group [12]. The dataset includes images of coronal slices stained for the immediate early gene c-Fos, taken from rats tested in the Helping Behavior Test (HBT, Fig. 2A). The HBT paradigm reflects prosocial motivation in rats who can release a trapped conspecific, thereby improving their well-being [13]. In the original study, rats selectively helped ingroup members (same-strain conspecifics) but not outgroup members (strangers of an unfamiliar strain, Fig. 2B), consistent with previous findings [14]. This dataset was originally manually quantified via visual identification of 84 ROIs, followed by ImageJ [15] intensity-based cell detection for region-by-region quantification (Fig. 2C, D).

Although this strategy has been helpful in elucidating the neural mechanisms of prosocial motivation, the analysis was very labor-intensive and used only a small fraction of the total available imaged brain tissue. Brainways was used to address these issues, resulting in a brain-wide quantification of c-Fos+ cell counts and enabling a re-analysis of neural activity patterns between rats under ingroup and outgroup conditions (Fig. 2E, F, see “Methods”). Overall, the re-analysis closely matched the original findings, providing strong evidence for the validity of this automated approach, as detailed below.

In addition, to directly compare the quantification produced with Brainways with the previously published manual quantification, we have conducted a second analysis that included only the slices and ROIs used in the original study (see Supplementary Note 2). This analysis replicated the previously reported findings while revealing several new significant ROIs. These additional findings may be due to (1) a more inclusive approach to tissue selection (i.e. analyzing all tissue in each slice rather than restricting to 250 μm boxes), (2) a more consistent registration and quantification pipeline provided by Brainways, mitigating inter-researcher variability that can arise during manual data collection, and (3) a misannotation or systematic bias within the Brainways quantification pipeline. To address this possibility, we conducted an additional visual inspection of the Brainways-derived registrations and include representative region segmentations along with c-Fos+ cell quantification results (Supplementary Fig. S2). These examples provide an overview of Brainways’ region segmentation and quantification performance. We also present a brief comparison of regions where Brainways and manual quantifications overlap or diverge (Supplementary Table S2).

**Fig. 3:**
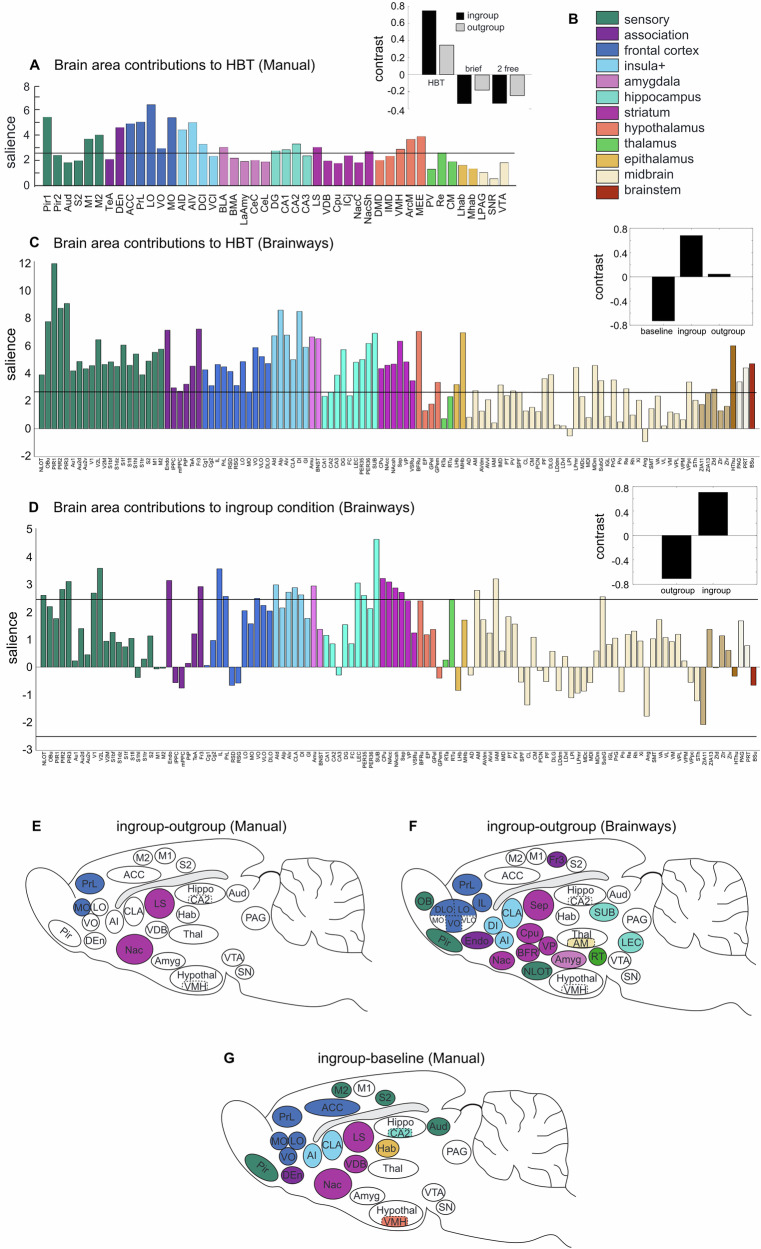
Comparison of manual and Brainways-assisted analysis of neural activity during the HBT reveals the pro-social brain network. **A** Partial least square (PLS) task analysis reveal a neural activity pattern unique to the HBT task (based on the manual acquisition, taken from Ben-Ami Bartal et al. (2021)). Salience represents the z score of boot-strapping tests, with regions that cross the black threshold lines significantly contributing to the contrast depicted in the inset (*p* < 0.01, black bars). Top-right: The black bar inset shows that HBT ingroup and outgroup conditions share a common neural activity pattern contrasting with the baseline conditions, which include a brief exposure to a trapped rat (“brief”) and exposure to a non-trapped rat separated by a wire mesh (“2 free”). **B** Color-coded legend of brain regions (**C**) brainways-assisted acquisition of the same data reveals a more detailed and significant neural activity pattern unique to the HBT task. Top-right: The black bar inset demonstrates a common neural activity pattern for HBT ingroup and outgroup conditions. However, here, the baseline conditions are grouped together into a single “baseline” category for clarity. **D** Contrasting the ingroup and outgroup conditions reveal a neural activity pattern unique to the ingroup condition. Top-right: The black bar inset demonstrates a neural activity pattern for the ingroup condition which contrasts with the outgroup condition. **E** Diagram of a rat brain showing regions significantly active (in color) for the HBT ingroup condition, derived using manual data acquisition, (**F**) or using Brainways-assisted acquisition. **G** The contrast comparing the ingroup and outgroup conditions using Brainways resembles the contrast comparing the ingroup and baseline conditions using manual acquisition. The complete abbreviation table is found in Table S3. (PLS figures are generated in Brainways in a single color; the coloring of the bars shown here was done using a Matlab script).

To identify patterns of neural activity associated with each condition, the neural activity of rats tested in the HBT conditions was compared to rats in the control conditions using multivariate task partial least squares analysis (PLS, see “Methods”, [16, 17]). The original analysis revealed a distinct neural network activated in the presence of a trapped ingroup member during the HBT (Fig. 3A, B). This network included the insula, ACC, orbitofrontal cortex, and sensory cortices. Importantly, activity in regions associated with the neural reward network, including NAC, LS, VDB, and parts of OFC and PRL, was specifically correlated with prosocial motivation and dissociated from arousal around a non-helped trapped outgroup member [12, 18].

Upon re-acquisition and analysis using Brainways, a similar significant latent variable (LV, *p* < 0.001) emerged, and following bootstrapping and permutations, provided a measure of the contribution of each brain region to the significant LV (termed ’salience’). This analysis revealed an increase in brain-wide activity for rats tested in HBT with trapped ingroup or outgroup members compared to rats in the baseline condition (Fig. 3C, black bars). This network included regions in the sensory cortex, frontal regions, and limbic and reward regions (Fig. 3C, colored bars). Notably, as previously reported, these regions include areas that participate in empathy in humans [19, 20] and rodents [21–24]. A direct comparison between the ingroup and outgroup conditions also produced a significant LV (LV1, *p* < 0.01), highlighting a distinct network that was significantly more active for ingroup members compared to outgroup members (Fig. 3D).

Next, the number of c-Fos+ cells was compared across the ingroup, outgroup, and baseline conditions (ANOVA, FDR corrected for multiple comparisons [25]). This analysis expanded on the original publication, where only four regions (MO, PrL, NAC, LS) reached the significance threshold for this comparison (Fig. 3E). Except for the MO, the three other regions (NAC, Septum, PrL) were replicated. Additionally, regions not previously quantified also proved significant, including the IL, VP, and parts of the thalamus (LEC, Subiculum, Fig. 3F). Multiple regions previously identified as active in the HBT ingroup condition compared to baseline were replicated (Fig. 3G), such as the claustrum, insula, piriform and endopiriform cortex, basal forebrain, Cpu, and the amygdala. Notably, while MO was not significant in the full analysis (*p* = 0.15), it reached significance in the analysis restricted to the original slices and ROIs (*p* = 0.02), suggesting that a specific subregion of MO may be involved in this behavior (see Supplementary Table S1 for the complete list of FDR‑corrected *p*‑values and  Supplementary Note 2). Thus, the Brainways-assisted reanalysis not only reproduced the original findings but also revealed a more extensive empathic helping network, providing further evidence for the role of this network in prosocial motivation.

#### Network Analysis

**Fig. 4:**
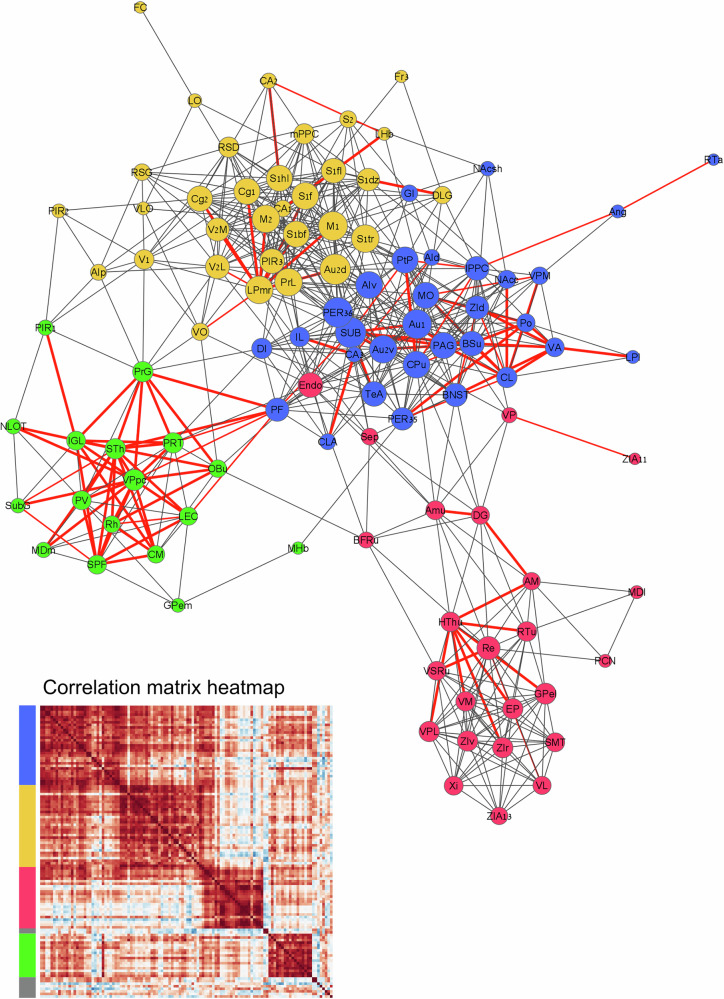
Network graph and corresponding interregion correlation matrix heatmap representing the functional connectivity between different brain regions in the ingroup HBT condition. Red edges denote statistically significant connections (*p* < 0.05) compared to a bootstrapped null network. Bolder lines indicate more significant *p* values. The visual rendering of the network graph was performed using Cytoscape (Shannon et al., 2003) based on a network graph file (*.graphml) produced in Brainways. The correlation matrix heatmap was generated via a python script and edited in Inkscape.

To examine the functional connectivity between different brain regions, network graphs based on interregional correlation matrices of c-Fos+ cell numbers were generated using Brainways based on a Pearson pairwise correlation matrix. The resulting graph was clustered to identify community structures and visualized using Cytoscape [26] (Fig. 4). The network graph shows a central cluster that includes multiple regions of the prosocial network (NAc, MO, Insula, IL, claustrum, and auditory sensory regions). A second cluster consists of frontal regions (RS, ACC, PrL, OFC, Fr3) and multiple regions in the visual and somatosensory cortex. This cluster also includes hippocampal regions (CA2, CA1, DLG) and the lateral habenula. The third and fourth clusters are primarily composed of thalamic subregions. This network, based on Brainways quantification, indicates that regions involved in prosocial motivation form a distributed brain-wide network connecting affective and motivational regions that are functionally connected during HBT [12]. In order to test the uniqueness of the connectivity to the task condition, we created a null-network distribution using a bootstrap analysis. We quantified the probability that each edge in the network was unique to the ingroup condition by comparing its correlation value to the distribution of the null network (see Supplementary Note 3). In each of the clusters, a subset of the edges was unique to the “ingroup” condition (*p* < 0.05, red edges in Fig. 4), whereas non-significant edges may represent general functional connectivity which is not task-specific. The significant edges provide potential candidates for future manipulation experiments.

### Mapping dopamine receptor activation in prosocial contexts using brainways and multiplex RNAscope

To further validate Brainways’ capabilities, we applied the software to a novel dataset using multiplex RNAscope imaging. This study aimed to investigate the recruitment of dopamine (DA) receptor-expressing cells during the HBT. The DA system plays a major role in social behavior, being a crucial part of the brain’s reward and motivation system and implicated in approach behavior [27, 28]. Specifically, neurons expressing D1 dopamine receptors (Drd1) are involved in the initiation of social interactions [29], whereas D2 dopamine receptors (Drd2) are implicated in pair-bonding formation in prairie voles [30]. In this study, we utilized whole-brain registration and quantification of cell type expression and activity markers, demonstrating Brainways’ ability to link specific cell types and their activity to task conditions. Specifically, we tested whether different populations of DA neurons were activated under different social conditions of the HBT.

**Fig. 5:**
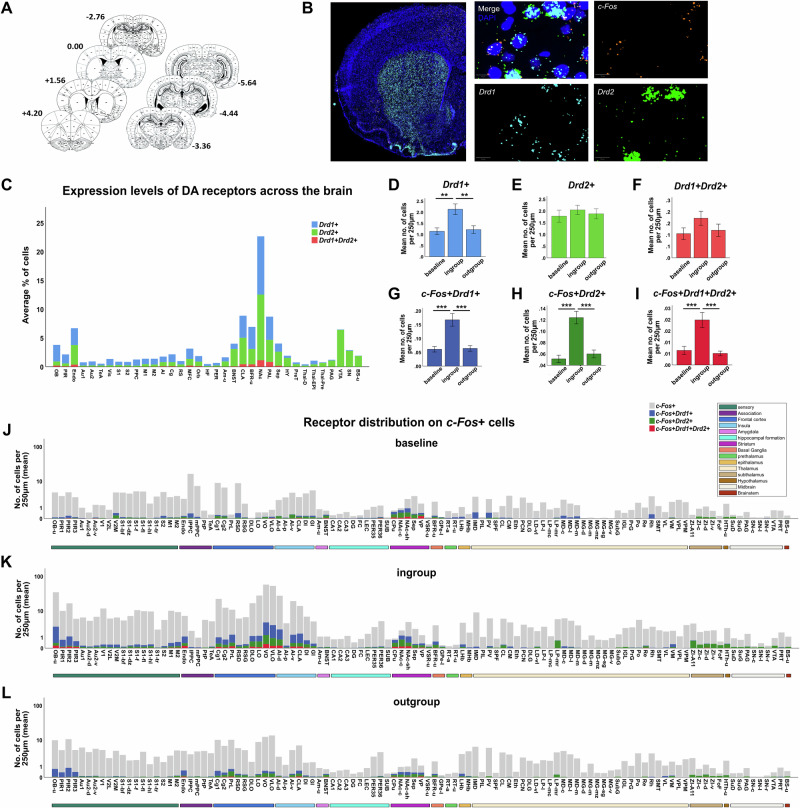
Receptor mapping on active cells during the “separated” HBT. **A** Illustration of the slices sampled from each brain for Multiplex RNAscope analysis staining. **B** Example of the histological images used for quantification of c-Fos, DrD1, and DrD2 mRNA co-labeling. **C** Mapping of DA receptors distribution across the brain. **D**–**I** Comparison of each receptor’s expression levels between the baseline, ingroup, and outgroup conditions of the HBT. Error bars represent SEM, **p < 0.01, **p < 0.001. **J**–**L** Summary of the identity of active (c-Fos + ) cells in each HBT condition. The complete abbreviation table is found in Table S3. (All graphs in this figure were generated via SPSS & Prism and edited in Inkscape).

Rats were tested in the “Separated” HBT paradigm, where they had no social contact after releasing a trapped conspecific from a restrainer. The sample included 7 coronal slices (Fig. 5A) obtained from 6 rats in the ingroup condition (3 males and 3 females) and 6 in the outgroup condition (3 males and 3 females). An additional 5 rats (3 females) served as the baseline group, not subjected to the HBT. Multiplex RNAscope was used to quantify co-labeled mRNA of c-Fos, Drd1, and Drd2 in the same sample (Fig. 5B), allowing for a detailed examination of receptor distribution within activated (*c-Fos* + ) cells.

Mapping of DA receptors, regardless of activation, was performed across the brain to validate the detection. Consistent with the literature, regions such as the striatum, nucleus accumbens, and basal ganglia showed high percentages of *Drd1+* and *Drd2+* cells (10-25%). Dopaminergic regions like the VTA and SN mainly expressed *Drd2*, while frontal and sensory regions predominantly expressed *Drd1*. A small population of cells co-expressed *Drd1* and *Drd2*, primarily in the basal ganglia, medial frontal cortex, and olfactory regions (Fig. 5C).

Brainways-assisted analysis revealed higher expression of *Drd1+* cells in the ingroup condition of the HBT compared to the baseline and outgroup conditions (F(2,1893) = 7.527, *p* < 0.001; Bonferroni, *p* = 0.003, *p* = 0.004, respectively; Fig. 5D). This effect was not observed in the *Drd2+* population (F(2,1893) = 0.389, *p* = 0.678) or in the *Drd1+Drd2+* population (F(2,1893) = 1.588, *p* = 0.21; Fig. 5E, F).

To test whether different populations of cells were activated in each HBT condition, we compared the number of cells co-labeled for *c-Fos* and DA receptors across the brain. We found higher activation of all subpopulations in the ingroup condition: *c-Fos+Drd1* + (F(2,1893) = 12.457, *p* < 0.001; Bonferroni, ingroup-baseline *p* < 0.001, ingroup-outgroup *p* < 0.001; Fig. 5G), c*-Fos+Drd2* + (F(2,1893) = 19.598, *p* < 0.001; Bonferroni, ingroup-baseline *p* < 0.001, ingroup-outgroup *p* < 0.001; Fig. 5H), and *c-Fos+Drd1+Drd2* + (F(2,1893) = 11.252, *p* < 0.001; Bonferroni, ingroup-baseline *p* < 0.001, ingroup-outgroup *p* < 0.001; Fig. 5I).

When testing for differences in specific subregions, although there is a trend of higher *c-Fos+Drd1+* cell numbers in sensory and frontal regions (Fig. 5J, K), these differences did not reach statistical significance. This lack of statistical significance could be due to the testing of complex behavior, which requires larger sample sizes to witness statistically significant differences. Despite this, the observed trends suggest that these regions may still play a role in the behavioral responses observed. Sensory and frontal regions showing a trend towards higher *Drd1* activation remain promising candidates for further pharmacological manipulation studies. Detailed investigation of these areas could reveal subtle but important roles in prosocial behavior. Therefore, future studies with larger sample sizes or more targeted manipulations are necessary to validate the involvement of these regions and elucidate their specific contributions to the neural networks underlying prosocial motivation.

In conclusion, this analysis highlights Brainways’ potential in neuropharmacological research by providing detailed neural insights and linking specific cell types to task conditions. Specifically, it demonstrated the sensitivity of the *Drd1+* subpopulation throughout the brain to the social identity of distressed conspecifics. The application of Brainways to the Multiplex RNAscope dataset successfully identified complex neural networks underlying prosocial behavior, showcasing the software’s capability for comprehensive and high-resolution analysis of brain-wide activity patterns.

## Discussion

In this paper, we introduce Brainways, an open-source tool for the automatic registration, quantification, and analysis of stained coronal brain slices. Brainways provides a comprehensive solution for researchers aiming to map brain-wide networks associated with various manipulations. By leveraging fluorescence tagging, Brainways enables detailed visualization and analysis of neural circuits, receptor mapping, population identification, and activity-identity relationships through co-labeling.

To demonstrate the utility of Brainways, we provide an analysis of two experiments. The first is a re-analysis of a previously published experiment initially examined using manual methods. Brainways-assisted quantification closely replicated the neural activity patterns identified in the original study and additionally uncovered regions not included in the manual quantification by increasing tissue coverage over a hundredfold. The second analysis applies Brainways to a novel dataset using Multiplex RNAscope imaging, effectively identifying neural networks underlying prosocial motivation in rats, linking specific cell types to task conditions. This dual demonstration of Brainways’ capabilities highlights its potential for high-resolution, brain-wide activity analysis, affirming its effectiveness and reliability across varied experimental contexts.

Brainways is optimized for analyzing coronal slices, a widely used brain imaging method in animal studies across various model organisms. This user-friendly tool facilitates rapid and comprehensive quantification of large datasets, making it particularly valuable for laboratories with limited programming expertise.

Brainways allows researchers to examine neural activity patterns associated with different task conditions by integrating several statistical analysis capabilities such as ANOVA, PLS, and graph network analyses. Using these capabilities, researchers can easily identify brain-wide neural networks related to an experimental condition, and central hubs for potential manipulations.

The innovative architecture of Brainways’ registration algorithm enhances its versatility, enabling the model to register brain slices from different species within a single framework. Trained on the Waxholm rat and Allen mouse atlases, Brainways is optimized for these species, but users can also manually register slices to any 3D atlas. We aim to expand support for additional species through community collaboration. Leveraging the Brainglobe API [31] as its backbone, Brainways provides out-of-the-box compatibility with numerous atlases, including those for zebrafish and humans.

To encourage further development in the field, Brainways is available as a fully open-source software. Researchers with no programming skills can use the software through a GUI, while those with programming expertise can utilize the Python API to integrate Brainways into other scripts or programs.

### Limitations

While the current approach is highly beneficial for rapidly achieving brain-wide quantification of fluorescence, there are some caveats to consider. The resolution provided by Brainways is influenced by two factors. First, the number of coronal slices provided by the user. As the number of sampled slices increases, so does the resolution of the resulting network. However, since Brainways significantly shortens the time from slide scanning to analysis, processing a higher number of slices per brain becomes much more feasible.

Another limitation is the level of parcellation offered by the atlas in use. For rats, the digital Waxholm Space Rat Atlas was used, and some key regions, such as the amygdala, hypothalamus, and basal forebrain, are not further subdivided. Fortunately, this atlas is under active development, and future versions are expected to include these important subregions. This issue does not affect investigators working with mice, as the existing Allen mouse brain atlas is highly parcellated.

Additionally, there is a tendency to underfit small parcellated subregions during the Non-rigid Registration phase. Therefore, users are advised to manually scan and correct fit errors during this phase, especially if focusing on differences between specific subregions of interest. To address this issue, we plan to train a non-rigid registration algorithm based on data collected using the Brainways GUI. It is important to note that, regardless of model accuracy, careful processing of the sections is essential for ensuring data quality in any software solution. To this end, the Brainways GUI is specifically designed to facilitate and expedite the visual scanning process and manual adjustment of the automatic registrations.

Furthermore, Brainways currently processes a single channel automatically, although co-labeling of several fluorescent markers is often utilized. Brainways supports importing cell detections of multiple channels, registering them to the atlas, and exporting co-labeling cell counts for each brain region and each animal in the experiment. Currently, Brainways only supports cell detection of a single stain from within the software, but we plan to include support for detecting multiple stains in the near future.

Brainways is designed for 2D histological sections and does not currently support 3D-to-3D registration pipelines commonly used for cleared brains with lightsheet imaging. Researchers working exclusively with volumetric lightsheet datasets should therefore rely on dedicated tools developed for full 3D registration. Incorporating volumetric 3D capabilities is an area of future development that we hope to explore.

We do not currently implement a specific chromatic aberration correction routine within Brainways; thus, users should address multi-channel alignment or aberrations with external software prior to registration.

While Brainways can incorporate multiple categorical behavioral parameters per subject, it does not currently integrate multidimensional behavioral parameters within a single statistical analysis; future developments aim to expand this feature to accommodate more complex behavioral profiles.

In addition, performing advanced network analyses of the type presented here currently requires familiarity with specialized network analysis tools and is not yet fully integrated into Brainways.

In conclusion, Brainways is a novel open-source AI-based software solution that can facilitate novel discovery of neurobiological mechanisms by providing quick and accurate automated network analysis from datasets of stained coronal slices. Brainways is freely available to the community as a fully open-source software, with the goal of advancing the field and promoting collaborative efforts for meta-analyses that reveal fundamental neural functions.

## Supplementary information

Supplementary Material

## Data Availability

All our code is publicly available on GitHub (https://github.com/bkntr/brainways). Data of cell counts per region that were used for the analyses are uploaded to the Open Science Framework depository (https://osf.io/fd8na/).
