# Supplementary material for: Mapping brain-wide activity networks: brainways as a tool for neurobiological discovery
Source: Neuropsychopharmacology. 2025 Apr 22;50(12):1885–95. doi: 10.1038/s41386-025-02105-3 (PMC12518845; doi:10.1038/s41386-025-02105-3)
Supplement: Supplementary file 1 — Supplementary Material [file 41386_2025_2105_MOESM1_ESM.docx]

**Mapping Brain-Wide Activity Networks:**

**Brainways as a Tool for Neurobiological**

**Discovery – Supplementary Information**

# Supplementary Note 1: Brainways’ modules

## Atlas Registration

The Atlas Registration module identifies the 3D location of a coronal brain slice within a reference atlas (Fig. 1D, left). It outputs the slice’s position on the Anterior-Posterior (AP) axis, its rotation in three axes, and the visible hemisphere. The input images, downscaled to capture rough features, are processed by a deep neural network trained to determine the AP axis. Users can manually adjust all registration parameters using sliders in the Brainways GUI, or access the algorithm via the Python API.

The Brainways GUI facilitates the registration of image slices to various atlases across species, using the Brainglobe Atlas API [1] for atlas retrieval and access. Since a rat atlas was unavailable in the Brainglobe repository, we added the Waxholm Space Rat Atlas version 4.0 [2,3]. Brainways supports automatic registration for the Waxholm Space Rat atlas and the Allen mouse brain atlas [4], and allows manual registration to any Brainglobe-available atlas, including those for zebrafish and humans.

To address the challenge of registering brain slices from different species within a single model, we developed a novel neural network architecture. Traditional models output the exact location of a slice within a specific atlas, limiting them to registering one species at a time. This made it impossible to register both rat and mouse brain slices to their respective atlases using a single model. Our new architecture, however, takes two images as input and outputs the position of one image relative to the other. This enables the model to be trained on pairs of rat slices and rat atlas images, as well as mouse slices and mouse atlas images, simultaneously. As a result, a single model can now register brain slices from different species.

During inference, the model receives pairs of images as input: one real brain slice and one atlas slice. The model outputs the position of the real slice relative to the atlas slice. By performing a binary search on the atlas slices, the model iteratively narrows down the location of the real slice within the atlas (Fig. S1). This procedure enables registration of the brain slice to the atlas, regardless of whether the slice is from a rat or a mouse.

The architecture is based on EfficientNet [5], with a linear head added to output the relative AP-axis position between the slices. The automatic registration algorithm was trained on over 700,000 mouse brain coronal slices from 8,164 publicly available datasets provided by the Allen Institute for Brain Sciences, which were registered to the Allen mouse atlas. Additionally, 2,697 rat brain slices from five different experiments performed by our group were registered to the Waxholm Space rat atlas and used to train the model for the rat atlas. For validation, 82 mouse datasets were used, with another 83 datasets set aside for testing. For the rat datasets, four were used for training and validation, with a random 10% of the images kept for validation, and one dataset was set aside for testing. To allow the model to register real slices to the atlas, random atlas slices were generated by virtually slicing the Waxholm Space rat atlas and the Allen mouse atlas. These synthetic slices were incorporated into the training process by randomly pairing synthetic or real slices as inputs for the model. This approach allowed the rat and mouse models to be trained and evaluated together within this unified framework.

To measure model performance, the AP value output from the network was compared with the AP value annotated by the expert annotators on the test dataset. If the AP value of the network matched the value of the annotator by up to 20 voxel units, the registration was considered correct. The mouse model achieved 98.1% accuracy according to this metric in the test dataset, with a mean absolute error of 5.01 voxels. The rat model achieved 92.0% accuracy, with a mean absolute error of 8.18 voxels.

## Rigid Registration

The main goal of the Rigid Registration module is to align the brain tissue in the input image with the corresponding atlas slice identified in the previous step (Fig. 1D, center). This module receives the coronal slice image and the matching atlas slice, and outputs four rigid registration parameters: left-right translation, up-down translation, horizontal scale, and vertical scale. Users can view and modify these parameters using sliders in the Brainways GUI, with the registration visualized by overlapping the input image with a transparent view of the atlas-defined brain regions.

Prior to rigid registration, the brain tissue is separated from the background to ensure only the tissue is registered. This is achieved by binarizing the input image using KMeans clustering on the pixel values, followed by connected component analysis on the binary image (Supplementary Note 3). A bounding box is then created around the brain tissue in both the input image and the atlas slice, and registration parameters are calculated to transform the input box to match the atlas box. The automatic Rigid Registration algorithm can be executed via a button in the Brainways GUI or through the Python API.

## Non-rigid Registration

Individual histological brain slices often exhibit significant deformations, unlike light-sheet imaging, where the whole brain is fixed in place. This issue arises due to the handling and accidental movement of the thin slices, usually between 20-60 µm. While experienced experimenters can minimize this problem, it remains a significant challenge in processing brain slice images.

The Non-rigid Registration module addresses this by receiving the brain slice image after rigid registration and the matching atlas slice, and outputs keypoints for elastic deformation using Thin Plate Spline (TPS) interpolation. This process is visualized by overlapping the input image with a transparent view of the brain regions as defined by the atlas.

To achieve elastic deformation, 24 initial source points are evenly spaced on the brain tissue, with 24 corresponding destination points placed at the same locations. These destination points can be adjusted using the Brainways GUI, resulting in the elastic deformation of the brain tissue to match the source points to the destination points (Fig. 1D, right). If the initial 24 point pairs are insufficient for registering key brain regions of interest, additional points can be added using the GUI.

Automatic Non-rigid Registration is facilitated using the TPS interpolation registration from the Elastix [6,7] Python package. Although Elastix performs well in matching the outlines of the brain slice to the atlas slice, it often struggles with more subtle internal structures. Therefore, Brainways allows experimenters to review and manually correct the registrations as needed. In future versions, we plan to train a deep neural network for TPS interpolation, which we expect to perform faster and better with sufficient training data.

## Cell Detection

The Cell Detection module quantifies the number of positively stained cells in each region. It processes brain slice images and outputs the x- and y-coordinates and the corresponding brain region for each positively stained cell or nucleus. This is achieved using an automatic cell detection algorithm, followed by registering the detected cells to the atlas using the previously computed registration parameters.

The StarDist [8] cell detection algorithm is employed for this purpose. StarDist generates a pixel mask for each detected cell (Fig. 1C), from which several parameters are extracted, including the central xand y-coordinates, the cell area in microns, and the mean intensity value. These parameters are used to filter out false positives. The filtered x- and y-coordinates are then transformed from the 2D image space to the 3D atlas space using the transformations computed in earlier modules. Finally, the corresponding brain region for each cell is determined based on its location in the atlas space.

It is recommended to train a custom cell detection model on your own data for optimal results. This can be done by training a custom StarDist model using a tutorial available in the Brainways documentation. Using a custom detection model is possible through selecting the relevant option in the Brainways GUI, allowing for better adaptability to unique datasets and improving detection accuracy.

To streamline the cell detection process, the Brainways GUI allows users to preview and adjust detection parameters before processing an entire dataset, saving time and resources. For those with existing cell detection pipelines, Brainways supports importing results from external software like QuPath or ImageJ. This feature is especially valuable for researchers who want to leverage Brainways’ registration and analysis tools without redoing cell detection.

Additionally, Brainways allows importing multi-stain cell detections, facilitating co-labelling analyses that can link cell types, activation states, and behaviors to specific brain regions, as demonstrated in Case Study #2. Future updates will include support for multiple stain detection within Brainways, further simplifying the analysis of complex datasets in a single platform.

## Analysis

After registration and cell counting, the results for the entire experiment (multiple subjects) are aggregated for statistical analysis. For each animal and brain region, the total area in microns, cell count, and normalized cell count (cells per 250*µm*^2^) are extracted. Normalized cell count is preferred for comparisons between animals as it accounts for variations in region areas between subjects. Detected cells across the whole brain can be viewed in 3D using the Cell Viewer module in the Brainways GUI (Fig. 1E, top-left) and exported to an Excel file for further analysis with external statistical software (Fig. 1F). The following analyses can be performed within Brainways on the registered cell counts.

***ANOVA contrast analysis.*** Brainways allows for ANOVA contrast analysis to identify and visualize brain regions that contribute to differences between experimental conditions (Fig. 1E, top-right). ANOVA is conducted on each brain region separately, followed by FDR correction for multiple comparisons [9]. Regions that significantly contribute to the contrast undergo post hoc analysis to identify specific differences between condition pairs.

***PLS contrast analysis.*** Task PLS is a multivariate statistical technique used to identify optimal neural activity patterns differentiating between experimental conditions [10,11]. PLS generates a set of mutually orthogonal pairs of latent variables (LV). One element of the LV represents the contrast, reflecting commonalities or differences between conditions. The other element, the relative contribution of each brain region (termed ’salience’), identifies regions that exhibit the activation profile across tasks, highlighting brain areas maximally expressed in a particular LV. Statistical assessment of PLS involves permutation testing for LVs and bootstrap estimation of standard error for brain region saliences. Latent variable significance is assessed via permutation testing, while salience reliability is evaluated using bootstrap standard error estimation. Brain regions with a bootstrap ratio >2.57 (approximately a 99% confidence interval) are considered to reliably contribute to the pattern. Missing values are interpolated by the average for the test condition.

The analysis results are exported to an Excel file and PNG files containing the salience plot, LV p-values, and LV contrast direction (Fig. 3C,D). Brainways uses the *pyls* Python package [12] to perform the PLS analysis.

***Network graph analysis.*** To examine the functional connectivity between the different brain regions, Brainways can be used to create a network graph based on interregion positive cell count correlation matrices (Fig. 1E, bottom-right). Network nodes consist of the different brain regions as defined in the atlas, and the edges of the network consist of significant correlations between the regions (the significance threshold can be adjusted by the user, set by default to p<0.05), based on Pearson’s pairwise correlation. The values are FDR corrected for multiple comparisons. The network graph is exported to a graphml file, and can be used with any graph analysis tools and algorithms for further analysis. See the Case Study section for an example analysis using the network graphs.

***Region-Specific Overview.*** After quantifying the entire experiment, Brainways offers a feature that allows users to select a specific brain region and view it across all brains in the experiment or within a specific experimental condition. This functionality enables researchers to quickly assess a particular brain region, facilitating the identification of consistent results, outliers, or issues with the quantification (Fig. 1E, bottom-left). This capability significantly enhances the efficiency of validating and interpreting experimental data.

# Supplementary Note 2: Direct Comparison of Brainways-Assisted and Manual Quantification Using the Same Slices and ROIs

We performed an additional analysis comparing ingroup, outgroup, and baseline conditions (ANOVA with post-hoc comparisons, FDR corrected for multiple comparisons [9]) that utilized exclusively the same slices and regions of interest (ROIs) used in the manually annotated dataset of our prior study (five slices per animal out of approximately 19 slices that were used in the full analysis). This analysis replicated the previously reported differences between ingroup and outgroup conditions in the PrL, MO, LS, and NAc-c (p<0.05; see Table S1 for a full list of p-values). NAc-sh trended higher in the ingroup condition, but this difference did not reach significance (p=0.17). In the original quantification, NAc was not parcellated for the analysis. As the full analysis had greater NAc coverage (including more than a single slice), the significance in NAc-sh emerged only in the full dataset. Additionally, we observed new significant differences in several other regions (BFR, CPu, DEn, LO, and Pir, p<0.05).

To further validate these findings and clarify the origins of discrepancies between manual and Brainways quantification, we include Table S2, which summarizes matches and mismatches across regions, and Figure S2, which provides representative examples of region segmentation and c-Fos quantification. Together, these additions illustrate the broader tissue coverage and consistent registration achieved by Brainways, supporting its accuracy and utility for c-Fos expression mapping.

# Supplementary Note 3: Bootstrap Analysis for Network Stability

We employed a bootstrap resampling procedure to rigorously evaluate the stability of the functional connectivity network derived for the ingroup HBT condition. First, we assembled an original dataset consisting of $N$ subjects, each with region-to-region correlation values. To generate each bootstrap replicate $b$ (where $b=1,2,\ldots,B$ and $B$ is user-defined), we sampled $N$ subjects with replacement from the original dataset. For each replicate, we computed a new adjacency matrix $A^{b}$, where each entry $A_{ij}^{b}$ was the correlation coefficient between region $i$ and region $j$ based on the resampled dataset. This process yielded $B$ distinct adjacency matrices that together characterize the empirical distribution of possible edge weights under repeated sampling.

Once the bootstrap distribution for each edge $\left( i,j \right)$ was obtained, we compared its average correlation in the target adjacency matrix, $A_{ij}^{\text{target}}$, to the set of bootstrapped correlation values $\left\{ A_{ij}^{b} \right\}$. We computed the p-value of the probability that a bootstrap-derived correlation was at least as large as the target sample’s correlation. Formally, for each edge $\left( i,j \right)$,

$p_{ij}=\frac{\sum_{b=1}^{B} \left[ A_{ij}^{b}\geq A_{ij}^{\text{target}} \right]}{B}$,

where $\left[ A_{ij}^{b}\geq A_{ij}^{\text{target}} \right]$ is an indicator function that equals 1 if $A_{ij}^{b}\geq A_{ij}^{\text{target}}$ and 0 otherwise. We then applied FDR multiple comparisons correction across all edges to control for inflation of Type I errors. Users can specify both the number of bootstrap iterations $B$ and the preferred correction method in the Brainways interface.

Edges with a significant p-value (e.g., $p_{ij}<0.05$ after correction) were considered robustly present in the network. For visualization, these edges were highlighted in red in the final network graph, with bolder lines indicating lower p-values. This representation allowed for immediate inspection of which brain region pairs demonstrated especially stable connectivity across replicates. The software stores the resulting statistical outcomes and adjacency matrices, enabling external follow-up analyses (e.g., in Cytoscape).

# Bibliography (Supplementary)

1. Claudi F, Petrucco L, Tyson A, Branco T, Margrie T, Portugues R. BrainGlobe Atlas API: a common interface for neuroanatomical atlases. JOSS. 2020;5:2668.

2. Papp EA, Leergaard TB, Calabrese E, Johnson GA, Bjaalie JG. Waxholm Space atlas of the Sprague Dawley rat brain. NeuroImage. 2014;97:374–386.

3. Osen KK, Imad J, Wennberg AE, Papp EA, Leergaard TB. Waxholm Space atlas of the rat brain auditory system: Three-dimensional delineations based on structural and diffusion tensor magnetic resonance imaging. NeuroImage. 2019;199:38–56.

4. Wang Q, Ding S-L, Li Y, Royall J, Feng D, Lesnar P, et al. The Allen Mouse Brain Common Coordinate Framework: A 3D Reference Atlas. Cell. 2020;181:936-953.e20.

5. Tan M, Le Q. EfficientNet: Rethinking Model Scaling for Convolutional Neural Networks. Proceedings of the 36th International Conference on Machine Learning, PMLR; 2019. p. 6105–6114.

6. Klein S, Staring M, Murphy K, Viergever MA, Pluim J. elastix: A Toolbox for Intensity-Based Medical Image Registration. IEEE Trans Med Imaging. 2010;29:196–205.

7. Shamonin D. Fast parallel image registration on CPU and GPU for diagnostic classification of Alzheimer’s disease. Front Neuroinform. 2013;7.

8. Schmidt U, Weigert M, Broaddus C, Myers G. Cell Detection with Star-Convex Polygons. In: Frangi AF, Schnabel JA, Davatzikos C, Alberola-López C, Fichtinger G, editors. Medical Image Computing and Computer Assisted Intervention – MICCAI 2018, vol. 11071, Cham: Springer International Publishing; 2018. p. 265–273.

9. Benjamini Y, Krieger AM, Yekutieli D. Adaptive linear step-up procedures that control the false discovery rate. Biometrika. 2006;93:491–507.

10. McIntosh AR, Bookstein FL, Haxby JV, Grady CL. Spatial Pattern Analysis of Functional Brain Images Using Partial Least Squares. NeuroImage. 1996;3:143–157.

11. Mcintosh AR. Mapping Cognition to the Brain Through Neural Interactions. Memory. 1999;7:523–548.

12. Markello R. pyls. 2023.

# Table S1: Comparison of full and matched analysis

| **Region** | **Full Analysis** | **Matched Analysis** |
| --- | --- | --- |
| **AI-d** | 0.011331274 | 0.298589637 |
| **AI-p** | 0.053579842 |  |
| **AI-v** | 0.018828649 | 0.061430326 |
| **AM** | 0.033819195 |  |
| **Am-u** | 0.011709681 | 0.068394348 |
| **Au1** | 0.870281724 |  |
| **Au2-d** | 0.311877101 | 0.373007162 |
| **Au2-v** | 0.745073578 | 0.453172412 |
| **BFR-u** | 0.035311976 | 0.03753961 |
| **BNST** | 0.231040753 |  |
| **BS-u** | 0.71359296 |  |
| **CA1** | 0.259808354 |  |
| **CA2** | 0.406240133 |  |
| **CA3** | 0.780392138 |  |
| **CLA** | 0.014595989 | 0.254872563 |
| **CPu** | 0.008159619 | 0.031977304 |
| **Cg1** | 0.954075186 | 0.707042347 |
| **Cg2** | 0.36425855 | 0.147167924 |
| **DG** | 0.139529026 | 0.181531337 |
| **DI** | 0.023240887 | 0.181531337 |
| **DLG** | 0.673433372 |  |
| **DLO** | 0.073835459 |  |
| **Endo** | 0.010586572 | 0.02793848 |
| **Fr3** | 0.01780807 |  |
| **GI** | 0.100893586 |  |
| **HTh-u** | 0.762945154 | 0.824587837 |
| **IL** | 0.005398275 |  |
| **LEC** | 0.039962484 |  |
| **LHb** | 0.398936987 |  |
| **LO** | 0.076710211 | 0.03753961 |
| **LP-mr** | 0.489114343 |  |
| **M1** | 0.954075186 | 0.452782766 |
| **M2** | 0.967822536 | 0.387615974 |
| **MD-m** | 0.559294851 |  |
| **MHb** | 0.166024206 |  |
| **MO** | 0.15305486 | 0.02793848 |
| **NAc-c** | 0.009763286 | 0.032100251 |
| **NAc-sh** | 0.014520652 | 0.174783054 |
| **NLOT** | 0.045912186 |  |
| **OB-u** | 0.046590346 |  |
| **PAG** | 0.33714263 |  |
| **PER35** | 0.062278654 |  |
| **PER36** | 0.131307113 |  |
| **PF** | 0.780392138 |  |
| **PIR1** | 0.103751381 | 0.680880449 |
| **PIR2** | 0.016219944 | 0.022153413 |
| **PIR3** | 0.011044089 |  |
| **PRT** | 0.667557518 |  |
| **PT** | 0.142619902 |  |
| **PV** | 0.133241595 |  |
| **PrL** | 0.02560432 | 0.024851347 |
| **PtP** | 0.929703203 |  |
| **RRe** | 0.566450789 |  |
| **RSD** | 0.540690701 |  |
| **RSG** | 0.573994338 |  |
| **RT-u** | 0.030421328 |  |
| **Re** | 0.240428813 |  |
| **S1-bf** | 0.247021877 |  |
| **S1-dz** | 0.396395658 |  |
| **S1-f** | 0.489114343 |  |
| **S1-fl** | 0.33643526 |  |
| **S1-hl** | 0.726734944 |  |
| **S1-tr** | 0.780392138 |  |
| **S2** | 0.292449685 | 0.205809118 |
| **SUB** | 0.030421328 |  |
| **Sep** | 0.016219944 | 0.024113979 |
| **TeA** | 0.55834809 |  |
| **V1** | 0.156068573 |  |
| **V2L** | 0.112551142 |  |
| **V2M** | 0.507153962 |  |
| **VLO** | 0.056494754 |  |
| **VO** | 0.041438733 | 0.066043012 |
| **VP** | 0.029203898 |  |
| **VSR-u** | 0.272394259 |  |
| **ZI-d** | 0.980306677 |  |
| **lPPC** | 0.681999594 |  |
| **mPPC** | 0.474671794 |  |

**Table S1.** FDR-corrected P-values of post-hoc comparisons between ingroup and outgroup conditions in the same samples analyzed either with the full dataset containing ~19 slices per rat with all available ROIs included (full analysis) or a subset matched to the originally published sample containing the same 84 ROIs in 5 slices per rat (matched analysis).

# Table S2: Summary of Matches and Discrepancies Between Brainways and Manual Quantification

| Region | Match? | Comment |
| --- | --- | --- |
| Pir | NO | Ingroup>outgroup in Brainways |
| Aud | YES |  |
| S2 | YES |  |
| M1 | YES |  |
| M2 | YES |  |
| TeA | YES |  |
| DEn | NO | Ingroup>outgroup in Brainways  Broader Endopiriform in 3D WHD Atlas |
| ACC | YES |  |
| PrL | YES |  |
| LO | NO | Ingroup>outgroup in Brainways  Brainways quantification seems correct upon visual inspection |
| VO | YES |  |
| MO | YES |  |
| AID | YES |  |
| AIV | YES |  |
| DCl | YES | CLA in WHS SD Atlas |
| VCl | YES | CLA in WHS SD Atlas |
| BLA | YES | Am-u in WHS SD Atlas |
| BMA | YES | Am-u in WHS SD Atlas |
| LaAmy | YES | Am-u in WHS SD Atlas |
| CeC | YES | Am-u in WHS SD Atlas |
| CeL | YES | Am-u in WHS SD Atlas |
| DG | YES |  |
| CA1 | YES |  |
| CA2 | YES |  |
| CA3 | YES |  |
| LS | YES | Sep in WHS SD Atlas |
| VDB | NO | Ingroup>outgroup in Brainways  Broader BFR-u region in 3D WHS Atlas |
| Cpu | NO | Ingroup>outgroup in Brainways  Much broader tissue, see figure |
| ICj | NO | Ingroup>outgroup in Brainways  Broader BFR-u region in 3D WHS Atlas |
| NacC | YES |  |
| NacSh | NO | There is no mismatch in the full analysis, only in the limited comparison. Brainways quantification seems correct upon visual inspection. |
| DMD | YES | HTh-u in WHS SD Atlas |
| IMD | YES |  |
| VMH | YES | HTh-u in WHS SD Atlas |
| ArcM | YES | HTh-u in WHS SD Atlas |
| MEE | YES | HTh-u in WHS SD Atlas |
| PV | YES |  |
| Re | YES |  |
| CM | YES |  |
| Lhab | YES |  |
| Mhab | YES |  |
| LPAG | YES | PAG in WHS SD Atlas |
| SNR | YES |  |
| VTA | YES |  |

**Table S2.** This table lists all regions included in the above comparison between Brainways-assisted and manual quantification. The “Match?” column indicates whether quantification results aligned across the two methods, and the “Comment” column provides additional context regarding observed discrepancies, including differences in parcellation, tissue coverage, and statistical outcomes.

# Table S3: Region abbreviation list

| **acronym** | **name** |
| --- | --- |
| AD | Anterodorsal thalamic nucleus |
| AId | Agranular insular cortex dorsal area |
| AIp | "Agranular insular cortex, posterior area " |
| AIv | "Agranular insular cortex, ventral area" |
| AM | Anteromedial thalamic nucleus |
| Amu | "Amygdaloid area, unspecified" |
| Ang | Angular thalamic nucleus |
| Au1 | Primary auditory area |
| Au2d | "Secondary auditory area, dorsal part" |
| Au2v | "Secondary auditory area, ventral part" |
| AVdm | "Anteroventral thalamic nucleus, dorsomedial part" |
| AVvl | "Anteroventral thalamic nucleus, ventrolateral part" |
| BFRu | "Basal forebrain region, unspecified" |
| BNST | Bed nucleus of the stria terminalis |
| BSu | "Brainstem, unspecified" |
| CA1 | Cornu ammonis 1 |
| CA2 | Cornu ammonis 2 |
| CA3 | Cornu ammonis 3 |
| Cg1 | Cingulate area 1 |
| Cg2 | Cingulate area 2 |
| CL | Central lateral thalamic nucleus |
| CLA | Claustrum |
| CM | Central medial thalamic nucleus |
| CNIC | "Inferior colliculus, central nucleus" |
| CPu | Caudate putamen |
| DG | Dentate gyrus |
| DI | Dysgranular insular cortex |
| DLG | Dorsal lateral geniculate nucleus |
| DLO | Dorsolateral orbital area |
| ECIC | "Inferior colliculus, external cortex" |
| Endo | Endopiriform nucleus |
| EP | Entopeduncular nucleus |
| Eth | Ethmoid-Limitans nucleus |
| FC | Fasciola cinereum |
| FoF | Fields of Forel |
| Fr3 | Frontal association area 3 |
| GI | Granular insular cortex |
| GPel | "Globus pallidus external, lateral part" |
| GPem | "Globus pallidus external, medial part" |
| HThu | "Hypothalamic region, unspecified" |
| IAM | Interanteromedial thalamic nucleus |
| IGL | Intergeniculate leaflet |
| IL | Infralimbic area |
| IMD | Intermediodorsal thalamic nucleus |
| IP | Interpeduncular nucleus |
| LDdm | "Laterodorsal thalamic nucleus, dorsomedial part" |
| LDvl | "Laterodorsal thalamic nucleus, ventrolateral part" |
| LEC | Lateral entorhinal cortex |

| **acronym** | **name** |
| --- | --- |
| LHb | Lateral habenular nucleus |
| LO | Lateral orbital area |
| LPl | "Lateral posterior thalamic nucleus, lateral part" |
| LPmc | "Lateral posterior thalamic nucleus, mediocaudal part" |
| LPmr | "Lateral posterior thalamic nucleus, mediorostral part" |
| lPPC | "Parietal association cortex, lateral area" |
| M1 | Primary motor area |
| M2 | Secondary motor area |
| MDc | "Mediodorsal thalamic nucleus, central part" |
| MDl | "Mediodorsal thalamic nucleus, lateral part" |
| MDm | "Mediodorsal thalamic nucleus, medial part" |
| MEC | Medial entorhinal cortex |
| MGd | "Medial geniculate body, dorsal division" |
| MGm | "Medial geniculate body, medial division" |
| MGmz | "Medial geniculate body, marginal zone" |
| MGsg | "Medial geniculate body, suprageniculate nucleus" |
| MGv | "Medial geniculate body, ventral division" |
| MHb | Medial habenular nucleus |
| MO | Medial orbital area |
| mPPC | "Parietal association cortex, medial area" |
| NAcc | "Nucleus accumbens, core" |
| NAcsh | "Nucleus accumbens, shell" |
| NLOT | Nucleus of the lateral olfactory tract |
| OBu | "Olfactory bulb, unspecified" |
| PAG | Periaqueductal gray |
| PaS | Parasubiculum |
| PCN | Paracentral thalamic nucleus |
| PER35 | Perirhinal area 35 |
| PER36 | Perirhinal area 36 |
| PF | Parafascicular thalamic nucleus |
| PIL | Posterior intralaminar nucleus |
| PIR1 | "Piriform cortex, layer 1" |
| PIR2 | "Piriform cortex, layer 2" |
| PIR3 | "Piriform cortex, layer 3" |
| Pn | Pontine nuclei |
| Po | Posterior thalamic nucleus |
| Pot | "Posterior thalamic nuclear group, triangular part" |
| PP | Peripeduncular nucleus |
| PrG | Pregeniculate nucleus |
| PrL | Prelimbic area |
| PrS | Presubiculum |
| PRT | Pretectal region |
| RT | Reticular (pre)thalamic nucleus |
| PT | Parataenial thalamic nucleus |
| PtP | "Parietal association cortex, posterior area " |
| PV | Paraventricular thalamic nuclei (anterior and posterior) |
| Re | Reuniens thalamic nucleus |
| Rh | Rhomboid thalamic nucleus |
| RRe | Retroreuniens thalamic nucleus |
| RSD | Retrosplenial dysgranular area |
| RSG | Retrosplenial granular area |
| RTa | "Reticular (pre)thalamic nucleus, auditory segment" |
| RTu | "Reticular (pre)thalamic nucleus, unspecified" |

| **acronym** | **name** |
| --- | --- |
| S1bf | "Primary somatosensory area, barrel field" |
| S1dz | "Primary somatosensory area, dysgranular zone" |
| S1f | "Primary somatosensory area, face representation" |
| S1fl | "Primary somatosensory area, forelimb representation" |
| S1hl | "Primary somatosensory area, hindlimb representation" |
| S1tr | "Primary somatosensory area, trunk representation" |
| S2 | Secondary somatosensory area |
| Sag | Nucleus sagulum |
| Sep | Septal region |
| SMn | Nucleus of the stria medullaris |
| SMT | Submedius thalamic nucleus |
| SNc | "Substantia nigra, compact part" |
| SNl | "Substantia nigra, lateral part" |
| SNr | "Substantia nigra, reticular part" |
| SPF | Subparafascicular nucleus |
| STh | Subthalamic nucleus |
| SUB | Subiculum |
| SubG | Subgeniculate nucleus |
| SuD | Deeper layers of the superior colliculus |
| SuG | Superficial gray layer of the superior colliculus |
| TeA | Temporal association cortex |
| V1 | Primary visual area |
| V2L | "Secondary visual area, lateral part" |
| V2M | "Secondary visual area, medial part" |
| VA | Ventral anterior thalamic nucleus |
| VL | Ventrolateral thalamic nucleus |
| VLO | Ventrolateral orbital area |
| VM | Ventromedial thalamic nucleus |
| VO | Ventral orbital area |
| VP | Ventral pallidum |
| VPL | Ventral posterolateral thalamic nucleus |
| VPM | Ventral posteromedial thalamic nucleus |
| VPpc | "Ventral posterior nucleus of the thalamus, parvicellular part" |
| VSRu | "Ventral striatal region, unspecified" |
| VTA | Ventral tegmental area |
| Xi | Xiphoid thalamic nucleus |
| ZIA11 | "Zona incerta, A11 dopamine cells" |
| ZIA13 | "Zona incerta, A13 dopamine cells" |
| ZIc | "Zona incerta, caudal part" |
| ZId | "Zona incerta, dorsal part" |
| ZIr | "Zona incerta, rostral part" |
| ZIv | "Zona incerta, ventral part" |


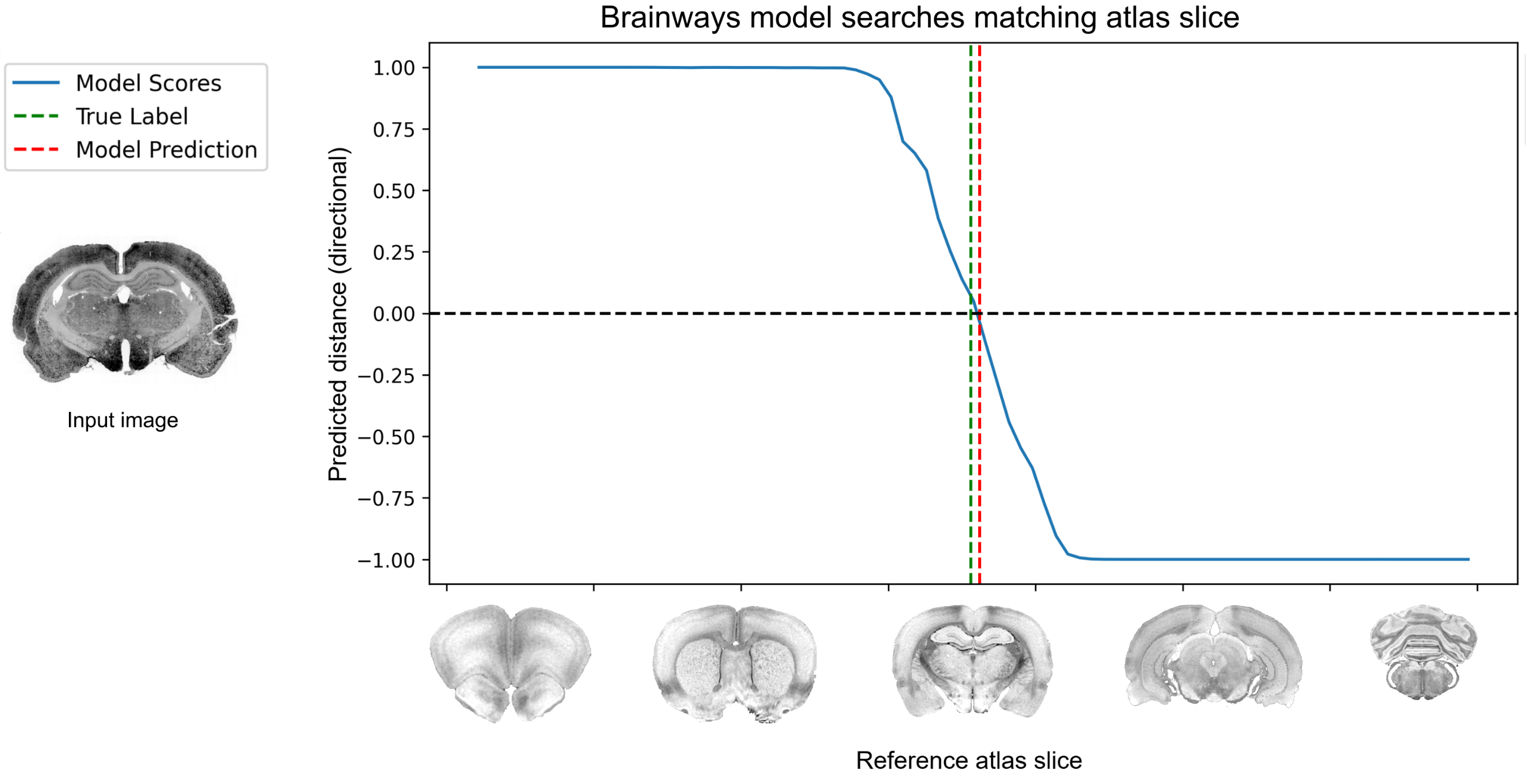


**Figure S1.** Brainways registration model uses binary search over atlas slices to find matching atlas slice. The Brainways registration model utilizes a binary search algorithm to identify the atlas slice that best matches a given input slice. This figure illustrates the predicted distance scores of a specific input slice when compared against slices from the entire atlas. The central plot shows the predicted directional distance score on the y-axis, ranging from -1.0 to 1.0, with the reference atlas slice on the x-axis, which represents all discrete slices available in the atlas. The step size along the x-axis is 25 microns. The blue curve represents the model scores, indicating how well each atlas slice matches the input slice. The green dashed line denotes the true label, and the red dashed line indicates the model’s prediction. At the bottom, a series of five reference atlas slices are shown for illustrative purposes only; they do not represent the only available positions. This highlights the model’s effectiveness in narrowing down the matching slice through iterative binary search, ultimately aiming for precise alignment between the input and atlas images.

# Figure S2: Representative Brainways-Based Region Segmentation and Quantification


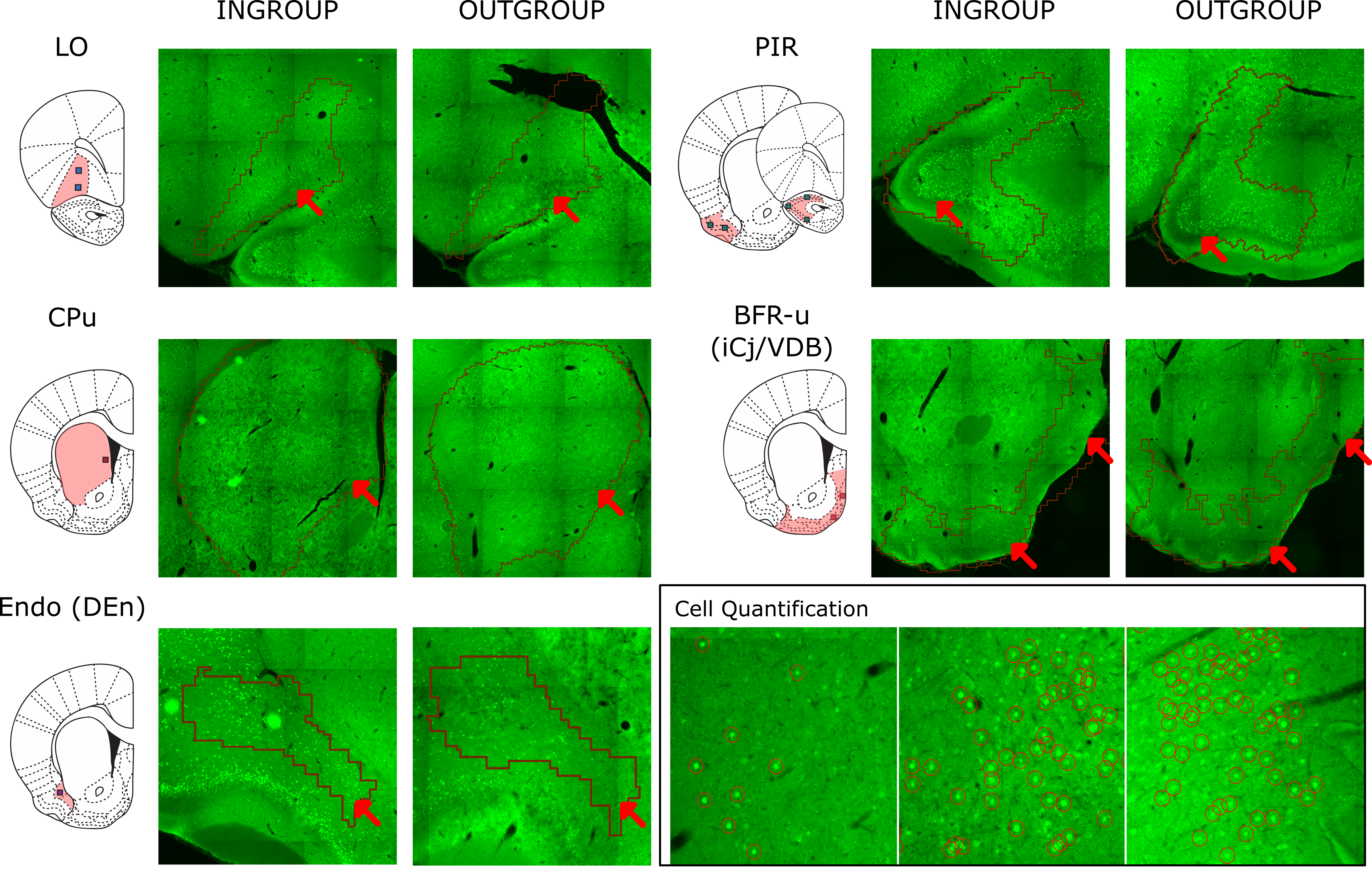


**Figure S2.** Representative Brainways-based region segmentation and quantification for regions that showed significantly greater activation in the ingroup condition compared to the outgroup condition, but did not show significant differences in the manual quantification. Left column: Coronal schematics showing both the 250 µm boxes used in the original manual quantification and the broader, pink-highlighted regions quantified by Brainways for the same ROIs. Where there are discrepancies between the Brainways and manual annotations due to atlas parcellation, the region title is displayed as Brainways (Manual). Middle column: Representative fluorescent images from rats in the ingroup condition. Right column: Representative fluorescent images from rats in the outgroup condition. The bottom-right panel provides a magnified view of c-Fos-positive cells (circled in red), illustrating how Brainways identifies and quantifies individual cells. These examples demonstrate (1) how Brainways segmentations can be visually confirmed, (2) that Brainways systematically covers a broader tissue area than the previous manual approach, and (3) higher c-Fos expression can be visually observed at regions Brainways identified as significantly more active in the ingroup condition.
